# Supplementary material for: The effect of the CONSORT statement on the amount of “unclear” Risk of Bias reporting in Cochrane Systematic Reviews
Source: PLoS One. 2020 Jul 10;15(7):e0235535. doi: 10.1371/journal.pone.0235535 (PMC7351499; doi:10.1371/journal.pone.0235535)
Supplement: Attachment — Submitted filename: Response to Reviewers.docx [file pone.0235535.s002.docx]

Dear dr. Berger,

We would like to thank you for your review of our work. We have read your papers with joy and interest , and have added the mentioned discussion points to the paper.

Please read our response to the points raised in the letter below.

1. Please ensure that your manuscript meets PLOS ONE's style requirements, including those for file naming
   a. We have revised the manuscript to adhere to the guidelines, we have substantially shortened the abstract
2. Please discuss why you have limited your search to systematic reviews published December, November and October 2016.
   a. In order to make our work feasible, we have limited our search to December, November and October 2016, we think this provides us with an extensive and representative sample of reviews.
3. We noticed you have some minor occurrence(s) of overlapping text with the following previous publication(s), which needs to be addressed:

https://doi.org/10.1371/journal.pone.0173358

https://doi.org/10.1136/bmjopen-2018-023729

https://doi.org/10.1016/j.jclinane.2017.01.017

In your revision ensure you cite all your sources (including your own works), and quote or rephrase any duplicated text outside the Methods section. Further consideration is dependent on these concerns being addressed.

a. We did not see the mentioned publications before, and after extensive reading, are unable to find the sentences in our paper. Could you give us an indication of the exact sentences?.

4. Please include captions for your Supporting Information files at the end of your manuscript, and update any in-text citations to match accordingly. Please see our Supporting Information guidelines for more information: <http://journals.plos.org/plosone/s/supportinxg-information>.
   a. We have included the captions for our supporting information file at the end of the manuscript.
5. You seem to have missed the opportunity to challenge the very notion of "unclear risk of bias". What does that mean, really? "Risk", by its very definition, conveys a lack of certainty. If we lack certainty about risk, then by definition there is risk. Unclear risk means there is a risk of a risk, which is to say, there is a risk. We are far too tolerant of authors who do not provide sufficient information to allow a reader to assess risk. The onus, or burden of proof, is on the authors to rule out risk. If they fail to do so, then they do NOT get the benefit of the doubt. That is the essence of high risk of bias. See:
   a. Thank you very much for your critical note. have now added this point in the discussion section of our paper.
6. Also, since you mention CONSORT so prominently, it seems fair to question what it offers, in terms of value added, since 1) it is grossly incomplete; 2) in some cases it is demonstrably wrong; and 3) even where it is correct all it does is repackage sage advice that was already out there prior to 1996. Adherence to CONSORT is neither necessary nor sufficient for a good trial, and you have the opportunity to mention that, too. See:
   a. We have revised the discussion in concern to this point.

We would like to thank you again for your thorough work. We hope you consider the revisions suitable. We hope you consider our manuscript suitable for peer-review.

Kind regards,

On behalf of all co-authors,

Maaike Rademaker
