# Supplementary material for: The effect of the CONSORT statement on the amount of “unclear” Risk of Bias reporting in Cochrane Systematic Reviews
Source: PLoS One. 2020 Jul 10;15(7):e0235535. doi: 10.1371/journal.pone.0235535 (PMC7351499; doi:10.1371/journal.pone.0235535)
Supplement: Attachment — Submitted filename: Response to Reviewers.docx [file pone.0235535.s003.docx]

Dear dr. Berger and reviewer,

Thank you in advance for the effort of reviewing our manuscript. We have revised it carefully and adjusted some items.

Please find our response to the commentary below.

Additional Editor Comments (if provided):

1. The revision looks much improved. Please address the reviewer comments, and also please note that the conclusions in the abstract are not, in fact, conclusions, since they are statements of what was directly observed. So this makes them results, and the question remains, what do you conclude from this study?

*We have updated the abstract.*

Reviewer #1: This is a relatively straightforward manuscript describing trends in time in the risk of bias in randomized clinical trials as determined by authors of Cochrane systematic reviews. The aim of the study was to evaluate the impact of the CONSORT reporting guidelines on the risk of bias as reported in these trials. The study was completed adequately, but there are a few questions about the methods that should be clarified in the manuscript.

1. Some justification needs to be included in the manuscript for why only the reviews published in December, November, and December 2016 were included. I see the authors responded to this question previously, but the reason needs to be included in the manuscript.

*We have added this to the discussion.*

2. Were the 989 RCTs unique. Sometimes RCTs are used in more than one review.

*Thank you for your point. We have checked this and adjusted in the review. There are 988 unique RCTS, one RCT was used for two reviews. Please see line 103. For the record both reviews judged the RoB for this RCT differently.*

3. Only one person extracted data. It would have been preferably for a second person to have extracted at least a sample.

*Thank you for your comment, we have checked 10% of the RCTs randomly with research randomizer.com. This resulted in 145 RCTs and 1015 RoB assesments, of which 19 missings. We were able to discover 4 mistakes data extraction for one RCT (0.39%). We corrected the data file, reran our analyses and updated the result section of the paper.*

This is especially concerning in that the methods state that “Data on sub categories was not assessed.” Often the risk of bias for blinding/masking is assessed by outcome. If a systematic reviewer assessed risk of bias for more than one outcome, how was the data extracted? Were all these ignored or was there some kind of averaging done?

*In this paper we stuck to the seven RoB items as defined by the Cochrane Risk of Bias tool. If a review adjusted the tool; we ignored the adjustments and stuck to the ‘pure’ seven RoB items. There was no averaging done. This was also added to the discussion.*

We would like to thank you both again for your revisions. We hope you consider the revisions suitable.

Kind regards,

On behalf of all co-authors,

Maaike Rademaker
